# MYB pathways that regulate UV-B-induced anthocyanin biosynthesis in blueberry (*Vaccinium corymbosum*)

**DOI:** 10.3389/fpls.2023.1125382

**Published:** 2023-01-30

**Authors:** Yan Song, Bin Ma, Qingxun Guo, Lianxia Zhou, Xintong Zhou, Ziqing Ming, Honglin You, Chunyu Zhang

**Affiliations:** Department of Horticulture, College of Plant Science, Jilin University, Changchun, China

**Keywords:** blueberry, MYB transcription factors, UV-B radiation, anthocyanin, universal stress protein

## Abstract

Ultraviolet-B (UV-B) promotes anthocyanin accumulation and improves fruit quality in plants. To explore the underlying network of MYB transcription factors that regulates UV-B-induced anthocyanin biosynthesis in blueberry (*Vaccinium corymbosum*), we analyzed the response of *MYB* transcription factor genes to UV-B treatment. Transcriptome sequencing analysis revealed that *VcMYBA2* and *VcMYB114* expression were upregulated and were positively correlated with the expression of anthocyanin structural genes under UV-B radiation according to weighted gene co-expression network analysis (WGCNA) data. The VcUVR8-VcCOP1-VcHY5 pathway perceives UV-B signals and promotes the expression of anthocyanin structural genes by upregulating *VcMYBA2* and *VcMYB114* or by regulating the VcBBXs-VcMYB pathway, ultimately promoting anthocyanin accumulation. By contrast, *VcMYB4a* and *VcUSP1* were downregulated under UV-B treatment, and *VcMYB4a* expression was negatively correlated with that of anthocyanin biosynthesis genes in response to UV-B. Analysis of *VcMYB4a*-overexpressing and wild-type blueberry calli exposed to UV-B radiation revealed that *VcMYB4a* represses UV-B-induced anthocyanin accumulation. Yeast one-hybrid and dual luciferase assays showed that the universal stress protein VcUSP1 directly bound to the promoter of *VcMYB4a*. These results suggest that the VcUSP1-VcMYB4a pathway negatively regulates UV-B-induced anthocyanin biosynthesis and provide insight into UV-B-induced anthocyanin biosynthesis.

## Introduction

Ultraviolet-B (UV-B) is a component of sunlight that affects the plant defense system, leading to increased accumulation of secondary metabolites, especially anthocyanin (Nguyen et al., 2017; Song et al., 2022). The UV-B-induced anthocyanin biosynthesis pathway is well characterized in many plants (Liu et al., 2018; Ding et al., 2021). In Arabidopsis (*Arabidopsis thaliana*), the UV-B photoreceptor UV RESISTANT LOCUS 8 (UVR8) interacts with CONSTITUTIVELY PHOTOMORPHOGENIC 1 (COP1) to activate UV-B signaling and initiate UV-B adaptation pathways in response to UV-B radiation (Oravecz et al., 2006; Rizzini et al., 2011). COP1 is a critical regulator of plant responses to UV-B that promotes flavonoid accumulation. COP1 activates ELONGATED HYPOCOTYL 5 (HY5, a bZIP transcription factor) and coordinates HY5-dependent and -independent pathways, ultimately resulting in UV-B tolerance (Oravecz et al., 2006; Bhatia et al., 2021). HY5 is a central transcription factor that promotes anthocyanin accumulation by regulating the expression of *MYB* transcription factors and anthocyanin biosynthesis genes (Stracke et al., 2010; Shin et al., 2013; An et al., 2017). B-box (BBX) proteins interact with HY5 or directly bind to the promoter sequences of *MYBs* or anthocyanin biosynthesis genes to regulate anthocyanin biosynthesis (Bai et al., 2019; Fang et al., 2019). Thus, *UVR8*, *COP1*, *HY5*, *BBXs*, *MYBs*, and anthocyanin biosynthesis genes form a UV-B-induced anthocyanin biosynthesis network and *MYBs* play an important role in this network.

MYB proteins are a superfamily of transcription factors that are responsive to one or multiple stress treatments and regulate the accumulation of secondary metabolites (Hartmann et al., 2005; Chen et al., 2006). In the R2R3-MYB gene family, subgroup 6 members promote anthocyanin biosynthesis and subgroup 4 members inhibit anthocyanin biosynthesis. For example, in the *Vaccinium* genus, *VbMYBA*, *VmMYBA1*, *VmMYBA2*, and *VcMYBA*, which belong to subgroup 6, activate anthocyanin biosynthesis, and, in strawberry (*Fragaria* × *ananassa*), *FaMYB1*, a member of subgroup 4, suppresses anthocyanin accumulation (Aharoni et al., 2001; Plunkett et al., 2018; Karppinen et al., 2021; Zhang et al., 2021). UV-B also affects the expression of *MYB* transcription factor genes from subgroups 4 and 6. In Arabidopsis, At*MYB75* from subgroup 6 is upregulated and *AtMYB4* from subgroup 4 is downregulated upon exposure to UV-B light (Jin et al., 2000; Tohge et al., 2011). In apple (*Malus domestica*), *MdMYB10* of subgroup 6 is upregulated throughout fruit development and influences anthocyanin production under UV radiation (Henry-Kirk et al., 2018). However, how MYB pathway genes function under UV-B radiation conditions merits further research.

Rich in anthocyanin compounds, blueberries (*Vaccinium corymbosum*) are often referred to as a “superfood” (Ribera et al., 2010; Norberto et al., 2013). The anthocyanin biosynthesis pathway in blueberry has been elucidated, and several structural genes associated with this pathway have been characterized (Zhang et al., 2017; Song et al., 2022). *VcMYBA* of subgroup 6 from blueberry has also been characterized; this transcription factor transactivates the *VcDFR* promoter to activate anthocyanin production (Plunkett et al., 2018). UV-B promotes anthocyanin accumulation in blueberry fruits and upregulates the expression of *VcBBX*, *VcMYB21*, *VcR2R3MYB*, and structural genes involved in anthocyanin biosynthesis (Nguyen et al., 2017). However, the UV-B-induced network underlying anthocyanin biosynthesis is unclear.

In this study, we identified MYB transcription factors that function in the response of blueberry to UV-B radiation based on transcriptome deep sequencing (RNA-seq) data and elucidated the network of transcription factors that positively regulates UV-B-induced anthocyanin biosynthesis according to weighted gene co-expression network analysis (WGCNA) data. In addition, analysis of blueberry calli overexpressing *VcMYB4a* and yeast one-hybrid and dual luciferase assays suggested the existence of a UV-B-induced anthocyanin biosynthesis network that is negatively regulated by VcMYB4a. Our findings broaden our understanding of UV-B-induced anthocyanin biosynthesis in blueberry.

## Materials and methods

### Plant materials and UV-B treatment

Wild-type (WT) and *VcMYB4a-*overexpressing blueberry calli (OE-1, OE-2, and OE-3 lines) were generated in our laboratory (Zhang et al., 2020; Yang et al., 2022).

All the calli were exposed to UV-B lamps (TL20/01; 311-nm Philips, Netherlands). WT calli were harvested to measure anthocyanin contents and WT and OE-1 calli were used to measure mRNA expression levels by reverse-transcription quantitative PCR (RT-qPCR) after 0, 6, 12, 24, and 48 h of UV-B radiation. The anthocyanin contents of WT and *VcMYB4a-*overexpressing calli treated with UV-B for 4 days were measured, using untreated calli as a control.

### Identification of transcription factor genes

RNA-seq data from calli treated for 0, 1, 3, 6, 12, and 24 h with UV-B radiation were downloaded from the BioProject database in the NCBI repository (https://www.ncbi.nlm.nih.gov/bioproject/PRJNA831018). The data were analyzed on the MKCloud online platform (https://international.biocloud.net). Using 0 h as a control, differentially expressed transcription factor genes from calli treated with 1, 3, 6, 12, and 24 h of UV-B radiation were identified according to the criteria of the absolute value of log_2_ (fold change) ≥ 1 and a false discovery rate (FDR) < 0.01 by the DEGSeq2 R package. Gene expression levels were quantified as fragments per kilobase of transcript per million fragments mapped (FPKM) values. The transcription factors were annotated using Protein family (Pfam) and Swiss-Prot databases (Apweiler et al., 2004; Finn et al., 2014).

### Cluster analysis of VcMYB transcription factors

The differentially expressed VcMYB transcription factors were obtained by deleting the redundant sequences. A phylogenetic tree was constructed from 52 VcMYBs of blueberry and 142 AtMYBs of Arabidopsis using the neighbor-joining method with the MEGA X program (Kumar et al., 2018). The phylogenetic tree was divided into different subgroups based on MYB transcription factors from Arabidopsis (Kranz et al., 1998; Dubos et al., 2010). The VcMYBs were named based on cluster and functional annotations from Swiss-Prot databases.

### Correlation analysis between *VcMYB4a* and genes involved in the UV-B-induced anthocyanin pathway

The differentially expressed genes from the WGCNA kMEblue module were selected to identify genes involved in the UV-B-induced anthocyanin pathway (Song et al., 2022). Pearson’s correlation coefficients (*r*) between the expression levels of differentially expressed genes were calculated using SPSS 19.0 software. Heatmap of UV-B-induced anthocyanin biosynthesis pathway genes and transcription factor genes were drawn using log_10_ (FPKM) values with TBtools software (v1.098761) (Chen et al., 2020).

### Reverse-transcription quantitative PCR

RT-qPCR analysis was performed for WT and *VcMYB4a-*overexpressing (OE-1) calli after 0, 6, 12, 24, and 48 h of UV-B radiation using an ABI 7900HT real-time PCR system. The relative expression levels of *VcPAL1*, *VcPAL3*, *Vc4CL2*, *VcCHS1*, *VcDFR*, *VcCHI3*, *VcF3H-1*, *VcF3H-2*, and *VcUFGT* genes were calculated using the 2^–ΔΔCt^ method and *VcGAPDH* was used as the reference gene. Primer sequences are shown in **Table S1**.

### Promoter analysis of *VcMYB4a* gene

The upstream sequences of *VcMYB4a* were cloned using a Genome Walking Kit (TaKaRa, Japan). The *cis*-elements of the *VcMYB4a* upstream sequence was analyzed for the presence of cis-acting regulatory sequences in the PlantCARE database (http://bioinformatics.psb.ugent.be/webtools/plantcare/html/).

### Yeast one-hybrid assays

The promoter fragment (1,718 bp) of *VcMYB4a* was inserted into the pHIS2 vector as bait (BD Biosciences, Shanghai, China). The *VcUSP1* (GenBank accession no. OP957062) sequence was recombined into the pGADT7 vector as prey (Clontech). The primer sequences are shown in **Table S1**. Yeast strain AY187 was co-transformed with different combinations of vectors and selected on SD medium lacking tryptophan and leucine. A series of 5-µL aliquots of 10× diluted yeast co-transformants were dropped onto SD medium lacking tryptophan, leucine, and histidine (SD/−Trp/−Leu/−His) and containing 10, 20, 30, 40, 50, and 75 mM 3-amino-1,2,4-triazole (3-AT), which was used to suppress the background histidine leakiness of the pHIS2 vectors. The co-transformed yeast was grown at 30°C for 3 days. Yeast cells harboring pHIS2-VcMYB4a/pGAD53m and pHIS2-p53/pGAD53m were used as negative and positive controls, respectively.

### LUC complementation imaging assay

The promoter fragment of *VcMYB4a* and CDS of *VcUSP1* were inserted into the pGreenII 0800-miRNA and Pcambia1302 vectors to construct the pVcMYB4a-LUC and GFP-VcUSP1 vectors, respectively (**Table S1**). Recombining vectors were co-injected into *Nicotiana benthamiana* leaves and luminescence was detected 48 h after infiltration with a live imaging apparatus. The ratio of Luc to Ren activity was determined using the Dual-Glo^®^ Luciferase Assay System (Promega). As a negative control, Pcambia1302 (GFP) and pVcMYB4a-LUC vectors were co-injected into the *N. benthamiana* leaves.

### Anthocyanin content measurement

The anthocyanin content of the samples was measured as described by Yang et al. (2022). Each sample (0.5 g) was soaked in 3 mL methanol with 1% HCl and incubated for 16 h in darkness at 4°C. After centrifugation, the supernatant was diluted with an equal amount of water and the absorbance of each diluted extract at 530 and 650 nm was measured with a spectrophotometer (UV-1600, Shimadzu). Total anthocyanin content was quantified using the equation A_530_-0.25×A_650_ (Rabino and Mancinelli, 1986).

### Statistical analysis

In the figures, each error bar represents the standard deviation (SD) of the mean of three independent biological replicates, and three technical replicates were performed for each biological replicate. Significant differences were detected using Tukey’s test, implemented in SPSS 19.0 software.

## Results

### UV-B radiation induces anthocyanin accumulation

**Figure 1:**
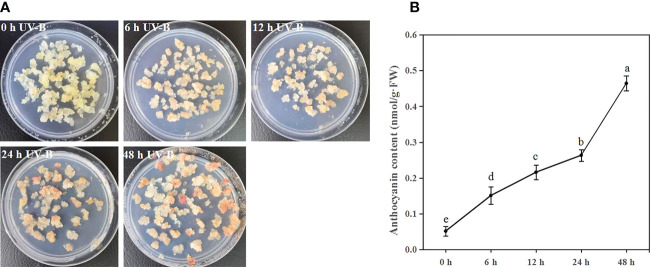
UV-B radiation induces anthocyanin accumulation in blueberry calli. The phenotype **(A)** and anthocyanin contents **(B)** of blueberry calli exposed to UV-B radiation for 0, 6, 12, 24, and 48 h. Error bars indicate ± SD of the mean of three independent biological replicates, each with three technical replicates; different letters indicate significant differences (*p* < 0.05) among samples by Tukey’s test.

To investigate the function of UV-B radiation on anthocyanin accumulation, blueberry calli were exposed to UV-B radiation for 0, 6, 12, 24, and 48 h. The proportion of callus that was red gradually increased during UV-B treatments (**Figure 1A**). Furthermore, the anthocyanin content significantly increased 2.9-, 4.2-, 5.1-, and 9.0-fold after 6, 12, 24, and 48 h of UV-B treatment relative to the 0 h treatment, respectively (**Figure 1B**). Thus, UV-B radiation promotes anthocyanin accumulation in blueberry calli.

### UV-B radiation regulates the expression of transcription factor genes

An analysis of Pfam and Swiss-Prot functional annotations revealed 370 transcription factor genes that were differentially expressed in response to UV-B radiation. The most highly enriched differentially expressed genes encoded MYB family members (23.77%), followed by AP2 (17.48%), WRKY (13.21%), bZIP (8.81%), and bHLH (6.92%) transcription factors (**Figure S1**; **Table S2**). After analyzing the sequences of *VcMYB* transcription factor genes and deleting the redundant *VcMYB* transcription factors, 52 *VcMYB* transcription factor genes were identified, namely 28 *R2R3-MYB*, 22 *1R-MYB*, 1 *3R-MYB*, and 1 *3R/4R-MYB* transcription factor genes (**Table S3**).

**Figure 2:**
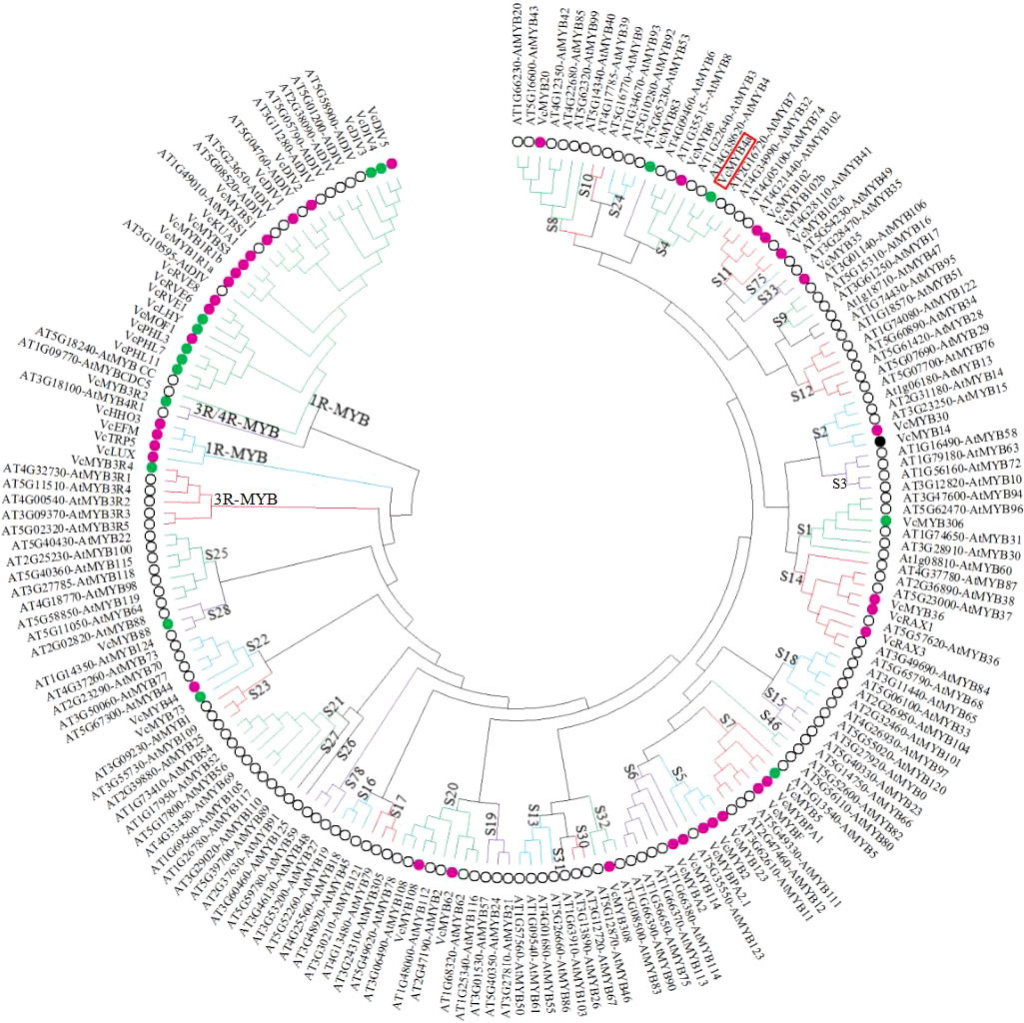
The 52 differentially expressed *VcMYB* transcription factor genes from blueberry and 142 *MYB* transcription factor genes from *Arabidopsis thaliana* were clustered into different subgroups using MEGA X with the neighbor-joining method. Magenta, green, and black dots represent upregulated, downregulated, and both up- and downregulated *VcMYBs* under UV-B radiation, respectively. Black circle represents *MYB* transcription factors from Arabidopsis. Red box indicates the position of *VcMYB4a*.

To predict the potential functions of the *VcMYBs*, phylogenetic analysis of the deduced protein sequences of 52 *VcMYB* and 142 *AtMYB* genes were performed (**Figure 2**). Of the 52 *VcMYB* genes, 28 *VcMYBs* from the R2R3-MYB subfamily belonging to 15 subgroups were upregulated and/or downregulated in response to UV-B radiation. *VcMYB114* and *VcMYBA2* were clustered in subgroup 6, and *VcMYB4a* was clustered in subgroup 4. Most genes from subgroup 6 promote anthocyanin accumulation, whereas most genes from subgroup 4 inhibit anthocyanin biosynthesis (Aharoni et al., 2001; Plunkett et al., 2018). *VcMYB114* and *VcMYBA2* expression increased and that of *VcMYB4a* decreased during UV-B treatment.

### *VcMYB4a* represses UV-B-induced anthocyanin accumulation

**Figure 3:**
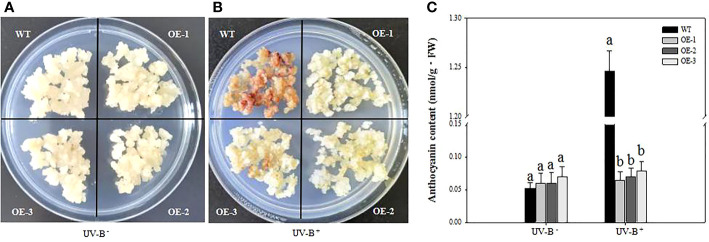
Anthocyanin contents of wild-type (WT) and *VcMYB4a-*overexpressing (OE-1, OE-2, and OE-3) blueberry calli after 4 days of exposure to UV-B radiation. Untreated calli **(A)** and calli after 4 days of UV-B treatment are shown **(B)**. **(C)** Anthocyanin contents of WT and *VcMYB4a-*overexpressing calli treated or not with UV-B radiation for 4 days. Error bars indicate SD of the mean of three independent biological replicates, each with three technical replicates; different letters indicate significant differences (*p* < 0.05) among samples by Tukey’s test.

To explore the role of *VcMYB4a* in the plant response to UV-B radiation, we treated three *VcMYB4a-*overexpressing lines (OE-1, OE-2, and OE-3) and wild-type (WT) calli with UV-B for 4 days and measured anthocyanin contents (**Figure 3**). In the absence of UV-B treatment, the anthocyanin content of *VcMYB4a-*overexpressing calli was not significantly different from that of WT calli (**Figure 3A**). Under UV-B treatment, anthocyanin rapidly accumulated in WT calli compared with *VcMYB4a-*overexpressing calli (**Figures 3B, C**). Thus, *VcMYB4a* was a repressor of UV-B-induced anthocyanin biosynthesis.

### *VcMYB4a* expression is negatively correlated with that of genes involved in UV-B-induced anthocyanin accumulation

**Figure 4:**
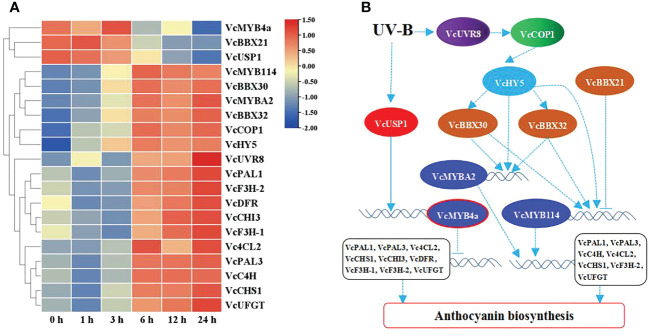
UV-B-induced anthocyanin biosynthesis pathway. **(A)** Heatmap representation of hierarchical clustering analysis of UV-B-induced anthocyanin biosynthesis pathway genes and transcription factor genes. Blue, low expression; red, high expression, based on log_10_(FPKM). **(B)** Proposed model of UV-B-induced anthocyanin biosynthesis pathways. Solid line indicates known signaling pathway; dotted line indicates putative signaling pathway. Arrows indicate activation; short lines indicate inhibition.

To reveal the role of *VcMYB4a* in the regulatory network underlying anthocyanin biosynthesis under UV-B radiation, we searched for genes that were co-expressed with *VcMYB4a* and involved in this network using the WGCNA package in R and calculated their Pearson’s correlation coefficients (*r*) (**Table 1**). The expression level of *VcMYB4a* was negatively correlated with that of *VcUVR8*, *VcCOP1*, and most genes involved in the anthocyanin pathway, including *VcPAL1*, *VcPAL3*, *Vc4CL2*, *VcCHS1*, *VcCHI3*, *VcDFR*, *VcF3H-1*, *VcF3H-2*, and *VcUFGT*. Both *VcMYB114* and *VcMYBA2* expression was positively correlated with the expression of *VcCOP1*, *VcHY5*, *VcBBX32*, and *VcBBX30* as well as that of the anthocyanin biosynthesis genes *VcPAL1*, *VcPAL3*, *VcC4H*, *Vc4CL2*, *VcCHS1*, *VcF3H-2*, and *VcUFGT*. However, *VcMYB114* expression was negatively correlated with *VcBBX21* expression. In a heatmap, *VcMYB4a* and *VcBBX21* were clustered together and were downregulated by UV-B radiation, whereas the other genes were upregulated by this treatment (**Figure 4A**; **Table S4**). These results indicate that *VcUVR8*, *VcCOP1*, *VcHY5*, *VcBBXs*, *VcMYBs*, and anthocyanin biosynthesis genes form a UV-B-induced anthocyanin biosynthesis network and that *VcMYB4a* is negative regulator of this network (**Figure 4B**).

**Table 1:** Pearson’s correlation coefficients (r) between the expression levels of genes from the UV-B-induced anthocyanin biosynthesis pathway.

Gene	*VcMYB4a*	*VcMYB114*	*VcMYBA2*	*VcHY5*
*VcUVR8*	−0.956**	0.725	0.954*	0.727
*VcCOP1*	−0.823*	0.934**	0.992**	0.916*
*VcHY5*	−0.659	0.926**	0.989*	1.000
*VcBBX21*	0.811	−0.92**	−0.894	−0.801
*VcBBX32*	−0.770	0.975**	0.995**	0.878*
*VcBBX30*	−0.717	0.990**	0.993**	0.967**
*VcPAL1*	−0.873*	0.883*	0.98*	0.713
*VcPAL3*	−0.832*	0.942**	0.981*	0.785
*VcC4H*	−0.810	0.926**	0.969*	0.746
*Vc4CL2*	−0.901*	0.923**	0.972*	0.811
*VcCHS1*	−0.822*	0.957**	0.99**	0.823*
*VcCHI3*	−0.862*	0.795	0.940	0.618
*VcDFR*	−0.817*	0.752	0.965*	0.515
*VcF3H-1*	−0.848*	0.726	0.946	0.517
*VcF3H-2*	−0.887*	0.832*	0.988*	0.641
*VcUFGT*	−0.87*	0.921*	0.965*	0.796

*Correlation significant at the 0.05 level. **Correlation significant at the 0.01 level.

### *VcMYB4a* represses the expression of anthocyanin biosynthesis genes in transgenic blueberry calli

**Figure 5:**
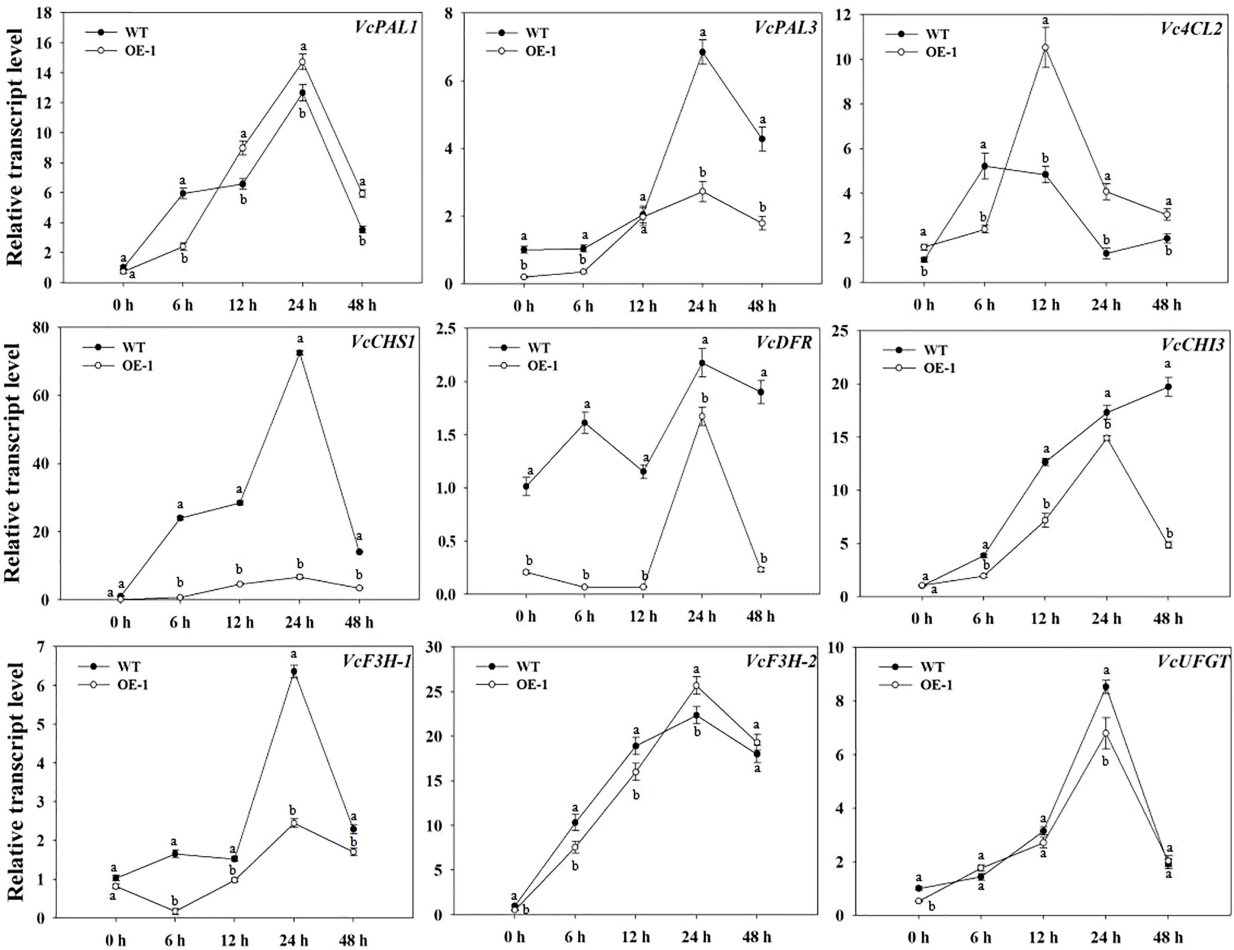
Expression of anthocyanin biosynthesis genes was repressed in the *VcMYB4a-*overexpressing blueberry calli (OE-1) compared with that in wild-type (WT) calli under UV-B treatment. Error bars indicate ± SD of the mean of three independent biological replicates, each with three technical replicates; different letters indicate significant differences (*p* < 0.05) among samples by Tukey’s test.

To identify potential downstream target genes of VcMYB4a, we compared the transcript levels of *VcPAL1*, *VcPAL3*, *Vc4CL2*, *VcCHS1*, *VcCHI3*, *VcDFR*, *VcF3H-1*, *VcF3H-2*, and *VcUFGT* between *VcMYB4a-*overexpressing calli (OE-1) and WT calli during UV-B treatment by RT-qPCR analysis (**Figure 5**). Overexpressing *VcMYB4a* in blueberry calli resulted in the downregulation of the anthocyanin biosynthesis genes *VcPAL3*, *VcDFR*, *VcF3H-2*, and *VcUFGT.* The transcript levels of *VcPAL1*, *VcPAL3*, *VcCHS1*, *VcDFR*, *VcF3H-1*, *VcF3H-2*, and *VcUFGT* genes rapidly increased and peaked after 24 h of UV-B radiation, and then decreased in both OE-1 and WT calli. For the same UV-B radiation time, the expression levels of *VcCHS1*, *VcDFR*, *VcCHI3*, and *VcF3H-1* genes in WT calli were higher than in OE-1 calli. These results suggest that *VcMYB4a* inhibits anthocyanin biosynthesis by downregulating anthocyanin biosynthesis genes.

### The upstream sequence of *VcMYB4a* is regulated by VcUSP1

**Figure 6:**
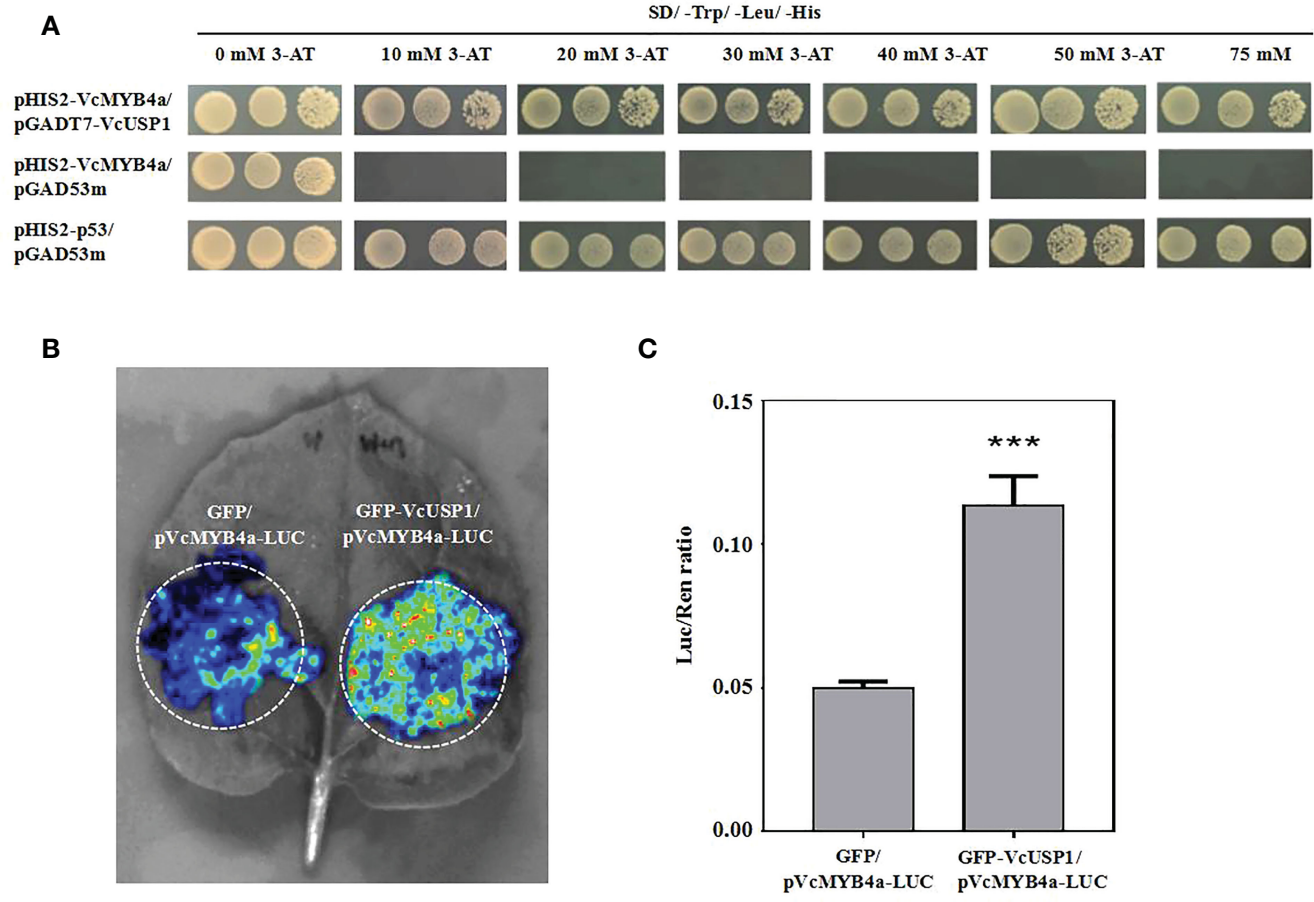
The VcUSP1 protein binds to upstream sequences of *VcMYB4a*. **(A)** Interaction of VcUSP1 with the *VcMYB4a* promoter in a yeast one-hybrid assay. pHIS2-VcMYB4a/pGAD53m was used as a negative control and pHIS2-p53/pGAD53m as a positive control. A series of 5-µL aliquots of 10 × diluted yeast cultures was grown on SD medium lacking tryptophan, leucine, and histidine (SD/–Trp/–Leu/–His) and with different concentrations of 3-amino-1,2,4-triazole (3-AT) for 3 days at 30°C. **(B)** Interaction of VcUSP1 with the *VcMYB4a* promoter using dual luciferase complementation imaging assays in *Nicotiana benthamiana* leaves. GFP/pVcMYB4a-LUC was used as a negative control. **(C)** The ratio of Luc-to-Ren activity was measured for the dual luciferase assay. Error bars indicate the SD of three biological replicates. The asterisks indicate statistically significant differences by Tukey’s test (***Correlation significant at the 0.001 level).

To elucidate the mechanism by which *VcMYB4a* suppresses anthocyanin metabolism under UV-B radiation, we isolated the promoter sequence of *VcMYB4a* from blueberry using a previously reported genome-walking protocol and predicted *cis*-elements using the PlantCARE database (**Tables S5; S6**). The main elements included light responsive elements, defense-related elements (drought and low-temperature), and phytohormone-related elements (particularly, those associated with methyl jasmonate, abscisic acid, and gibberellin). We cloned the 1,718-bp sequence upstream of the coding region of *VcMYB4a* and inserted it into the pHIS2 vector. We then performed a yeast one-hybrid assay to identify proteins that interact with the *VcMYB4a* promoter sequence from a yeast library. The universal stress protein VcUSP1 directly bound to the promoter region of *VcMYB4a* (**Figure 6A**). To validate the result of the yeast one-hybrid assay, we performed luciferase complementation imaging assays in *N. benthamiana* leaves. Co-expression of GFP-VcUSP1 with pVcMYB4a-LUC led to a clear increase in LUC signal and Luc/Ren ratio compared with leaves co-expressing the control vectors (**Figure 6B**; 6C). These data confirmed that *VcUSP1* directly bound to the promoter region of *VcMYB4a* to regulate its expression.

## Discussion

UV-B radiation promotes anthocyanin accumulation *via* the UVR8-COP1-HY5 pathway (Rizzini et al., 2011; Bhatia et al., 2021). The HY5 transcription factor directly binds to upstream sequences of *MYB* transcription factor genes to promote their expression (Stracke et al., 2010; Shin et al., 2013; An et al., 2017). BBX proteins directly interact with HY5 to enhance its binding to the promoters of *MYBs* or regulates the expression of *MYBs* to promote anthocyanin accumulation. However, B-box protein also suppresses anthocyanin accumulation by interacting with HY5 to synergistically inhibit the expression of *MYBs* (Bai et al., 2014; An et al., 2019; Fang et al., 2019). In the current study, we established that *VcUVR8*, *VcCOP1*, and *VcHY5* are upregulated under UV-B treatment, and identified a positive correlation between *VcHY5* and *VcCOP1* expression (**Figure 4A**; **Table 1**). Furthermore, *VcHY5* expression was found to be positively correlated with *VcBBX30*, *VcBBX32*, *VcMYB114*, and *VcMYBA2* expression. These results suggest that VcUVR8 perceives UV-B radiation and then upregulates *VcCOP1* and *VcHY5*. VcHY5 then combines with VcBBX32 and VcBBX30 to promote *VcMYB114* and *VcMYBA2* expression. Therefore, *VcHY5*, *VcBBX32*, *VcBBX30*, *VcMYB114*, and *VcMYBA2* form a positive regulatory network of anthocyanin biosynthesis under UV-B treatment (**Figure 4B**).

Transcription factors play essential roles in plant responses to abiotic stress (Chen et al., 2010; Seo and Park, 2010). UV-B radiation regulates the expression of most transcription factor genes, including MYB, bHLH, WRKY, NAC, and bZIP family genes (Ma et al., 2020; Ding et al., 2021). The current results support this finding. To date, 229 non-redundant MYB sequences have been identified in the blueberry genome, including 72 that respond to drought treatment in leaves and 69 in roots (Wang et al., 2021). In this study, 52 non-redundant MYB transcription factor genes from the R2R3-MYB, 1R-MYB, 3R-MYB, and 3R/4R-MYB subfamilies were found to function in the response of blueberry to UV-B radiation. Fifteen subgroups of R2R3-MYB subfamily members are involved in the response to UV-B radiation, as they were upregulated or downregulated by this treatment (**Figure 2**). These results improve our understanding of the roles of blueberry MYB TFs in response to abiotic stress.

R2R3-MYB transcription factors from subgroup 6 control anthocyanin biosynthesis by regulating the expression of structural genes involved in this process. For example, AtMYB113, AtMYB114, AtMYB75, and AtMYB90 promote anthocyanin biosynthesis by upregulating the expression of late anthocyanin structural genes in Arabidopsis (Teng et al., 2005; Gonzalez et al., 2008). Blueberry (*Vaccinium* section *Cyanococcus*) expressing *MYBA* of subgroup 6 activated anthocyanin production in *N. benthamiana*, and MYBA transactivated the *DFR* promoter from blueberry and other species (Plunkett et al., 2018). In the current study, we identified two *VcMYB* genes of subgroup 6, *VcMYB114* and *VcMYBA2*, whose expression was positively correlated with that of structural genes involved in anthocyanin biosynthesis that were upregulated under UV-B radiation (**Table 1**; **Figure 4**). Thus, *VcMYB114* and *VcMYBA2* function as activators in the UV-B-induced anthocyanin biosynthesis network by promoting downstream gene expression.

VcMYB4a, belonging to subgroup 4, inhibits lignin biosynthesis and negatively regulates plant responses to salt, drought, and temperature stress (Zhang et al., 2020; Yang et al., 2022). Our study showed that the expression levels of *VcMYB4a* were negatively correlated with those of anthocyanin biosynthesis genes. Thus, *VcMYB4a* likely encodes an inhibitor of the anthocyanin biosynthesis pathway. Yeast one-hybrid and dual luciferase assays showed that VcUSP1 binds to the promoter region of *VcMYB4a* and directly regulates its expression (**Figure 6**). USP is a ubiquitous protein that plays an important role in abiotic stress tolerance in plants (Jung et al., 2015). Some USP proteins enhance osmotic stress, salinity, drought, cold, freezing, or heat tolerance in plants (Loukehaich et al., 2012; Jung et al., 2015; Udawat et al., 2016; Guo et al., 2020). However, a recent study showed that *AtUSP17*, a multiple stress-inducible gene, encodes a universal stress protein in Arabidopsis that negatively regulates salt tolerance by modulating multiple signaling pathways (Bhuria et al., 2022). In this study, *VcUSP1* was found to be downregulated under UV-B treatment (**Figure 4A**). These findings suggest that *VcUSP1* and *VcMYB4a* form a negative regulatory network and that UV-B radiation downregulates the expression of *VcMYB4a* by repressing *VcUSP1* expression.

## Data availability statement

The datasets presented in this study can be found in online repositories. The names of the repository/repositories and accession number(s) can be found in the article/**Supplementary Material**.

## Author contributions

CZ conceived and designed the project. YS, BM, QG, LZ, XZ, ZM and HY participated in the experiments and analyzed the data. YS and BM drafted the manuscript. CZ modified the manuscript. All authors read and approved the final manuscript. All authors contributed to the article and approved the submitted version.

## Supplementary material

Supplementary Figure 1 Various transcription factor families were induced by UV-B radiation in blueberry callus. Transcriptome deep sequencing (RNA-seq) data were obtained for blueberry callus treated with UV-B radiation for 0, 1, 3, 6, 12, and 24 h. MYB, MYB proteins; bHLH, basic helix-loop helix; WRKY, WRKY proteins; NAC, NAC domain–containing protein; bZIP, basic region/leucine zipper; GATA, GATA zinc finger; AUX/IAA, auxin-responsive protein; HSF, heat stress transcription factor; Auxin, auxin response factor; DOF, Dof zinc finger protein; AP2, AP2 domain–containing protein.
