# Supplementary figures and images for: MYB pathways that regulate UV-B-induced anthocyanin biosynthesis in blueberry (Vaccinium corymbosum)
Source: Front Plant Sci. 2023 Jan 30;14:1125382. doi: 10.3389/fpls.2023.1125382 (PMC9923047; doi:10.3389/fpls.2023.1125382)

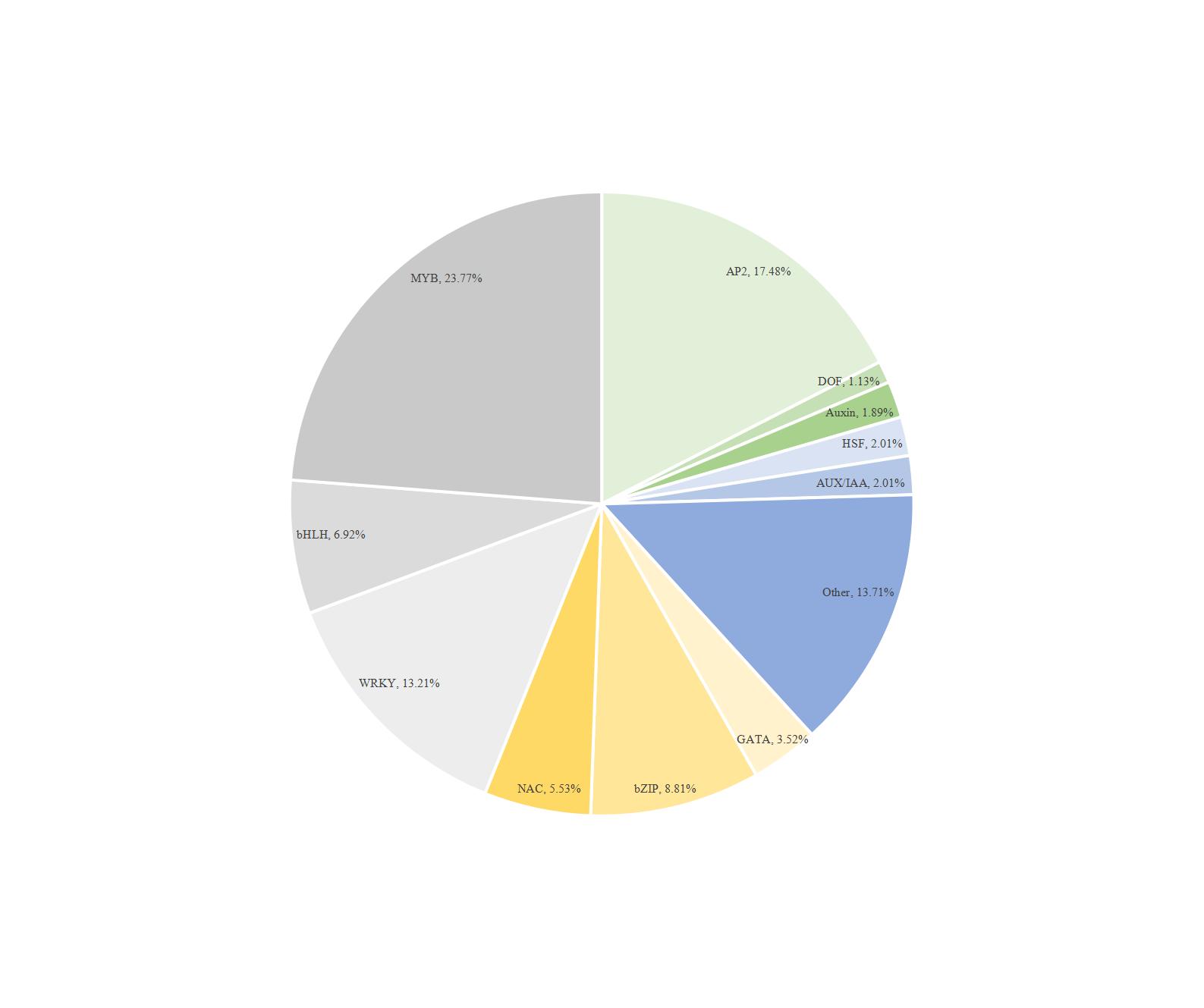

Supplement: Supplementary Figure 1 — Various transcription factor families were induced by UV-B radiation in blueberry callus. Transcriptome deep sequencing (RNA-seq) data were obtained for blueberry callus treated with UV-B radiation for 0, 1, 3, 6, 12, and 24 h. MYB, MYB proteins; bHLH, basic helix-loop helix; WRKY, WRKY proteins; NAC, NAC domain–containing protein; bZIP, basic region/leucine zipper; GATA, GATA zinc finger; AUX/IAA, auxin-responsive protein; HSF, heat stress transcription factor; Auxin, auxin response factor; DOF, Dof zinc finger protein; AP2, AP2 domain–containing protein. [file Image_1.jpeg]
